# Deborah Chambers and Pablo Gracia, *A Sociology of Family Life*

**DOI:** 10.1007/s10680-023-09672-5

**Published:** 2023-07-19

**Authors:** Alina Pelikh

**Affiliations:** Centre for Longitudinal Studies, University College London, 55-59 Gordon Square, London, WC1E 6BT UK

The diversity and complexity in partnership and family behaviours have increased in recent decades. The book “A sociology of family life”, written by Deborah Chambers and Pablo Gracia makes a significant contribution to the field of family sociology and demography by effectively summarizing the current state of affairs from a global lens, pointing out the existing limitations and suggesting directions for future research. The book presents multiple theoretical considerations and challenges and is rich in empirical evidence.

The book has three major aims as outlined by the authors in the Introduction: (1) “to document and analyse the growing diversity in personal and family life”; (2) “to study the intersections of gender, sexuality, social class, race and ethnicity to assess how these social factors frame personal and family life”; and 3) “to study changing families and intimacies through a global lens”.

The volume is organized into 9 chapters. Chapters 1 and 2 outline the key approaches to family and kinship studies from the late nineteenth century until the present day. The authors summarize the history of theoretical developments from the sole focus on biological ties to the recognition of the importance of social ties. The first chapter also draws on the seminal work of Talcott Parsons on “functional families” (1956), whose key purposes were “primarily socialization and the stabilization of adult personalities”, which could be achieved with the specialization of roles between husbands and wives as breadwinners and housewives (the “Golden Age” of the American family). However, the rapidly changing times both in terms of economic and societal developments posed a number of challenges to this mainstream ideology of a “traditional family”. The authors present multiple criticisms of this approach including the lack of acknowledgement of the changing gender roles and rising independence of women, as well as the blindness towards existing racial and ethnic inequalities. Chapter 2 continues with the transformation of theoretical approaches to studying families introduced in the 1990s. These theories are centred around the concept of individualization of the life course, which implies greater autonomy around individuals’ life choices (Beck, 1992; Beck & Beck-Gernsheim, 1995, 2002; Giddens, 1992). Applied to family and partnership behaviours, individualization puts a greater emphasis on emotional interaction and trust as the pillars of a “pure partnership” which lasts as long as there is a mutual understanding and agreement between the partners (“confluent love”). The individualization thesis is a powerful concept often used in family studies to explain the growing diversity in new partnership and family behaviours, including the rise in cohabitation and living-apart-together relationships (LAT) as well as the diversity in LQBTQ+ experiences. Its main limitation, however, includes the lack of attention to structural inequalities in terms of gender, race, sexual orientation, and social class. In response, the chapter discusses new approaches focusing on family practice and family display (i.e. how family lives are practiced, experienced, and expressed), which puts greater emphasis on social interactions as well as cultural and religious customs and practices.

Chapters 3 and 4 address some of the major shifts in parent–child relationships including changes in parenting values and practices as well as the debate around children’s agency and rights. The authors describe the shift towards “intensive” parenting ideology based on child-centred values. This shift involved a transformation of norms around motherhood and fatherhood which implied a greater involvement of both parents in children’s educational and leisure activities. However, despite the increased involvement of fathers, the majority of caring tasks still disproportionately lie on mothers, who have to juggle between work and family life, often leading to women’s withdrawal from the labour market. These issues are discussed in relation to parental leave and childcare provision across various institutional settings. Moreover, the authors highlight the often-neglected diversity among ethnic minorities’ parenting practices. Following the rise in divorce and separation in the past few decades, these chapters also draw attention to new challenges associated with co-parenting after separation as well as changing attitudes towards lone motherhood and its implications for children.

Chapter 5 discusses the changing dynamics of ageing, including changes in living arrangements, intergenerational transfers, and governmental provision of pensions, and spending on care across various institutional settings. The authors discuss the increased heterogeneity among groups of older people including the rise of single-person and same-sex couple households and new societal challenges associated with it. The chapter highlights the increased significance of friendships in ageing (“chosen families”) which are particularly important among LGBTQ+ communities, as well as single, widowed, or childless people. The authors also address the cultural differences in attitudes and norms towards ageing and multigenerational living arrangements. They describe how these relationships are undergoing great adjustments in migrant families, including those who leave their elderly family members behind in the countries of origin. Following up on this, Chapter 6 continues with the discussion of issues around migration and globalization and its consequences for intimate relationships, marriages, and family life. In particular, the authors focus on the flow of female migrants from low-income countries who leave their families behind to take up caring jobs in high-income countries. The chapter also discusses the transformation of the tradition of arranged marriages as well as the growing practice of “mail-order brides”. Chapter 7 demonstrates a series of historical case studies of family planning policies from Japan, Romania, India, and China.

Chapter 8 draws attention to the rapidly developing new reproductive technologies (e.g. IVF or donor insemination) and how it changes the way families are created. The authors discuss various ethical and legal issues related to donor conceptions and anonymity, including birth certificate registration and new questions arising around the meaning of biological and social parenthood and the future wellbeing of children. The chapter provides a rigorous overview on globalization and commercialization of infertility treatments and its consequences, including issues around legality and the price of surrogacy across different contexts. The authors also discuss inequalities in access to treatments, including both financial and legal barriers, especially in relation to LGBTQ+ couples and single people.

The final chapter (Chapter 9) concludes with a discussion of the need for new approaches in family sociology research, which would incorporate new concepts of intimacies and family practice (e.g. “families by choice” and LGBTQ+ intimacies), and consider how these new concepts are shaped by global economies.

The book presents a well-rounded and up-to-date discussion of the growing diversity in personal and family life across the globe and its consequences. Gender, racial, and class inequalities lie in the heart of these discussions. The authors challenge the western-centric concerns that the modern world is experiencing a crisis of commitment and care by providing rich empirical evidence of why this is not the case. Each chapter contains an introduction with a short summary as well as conclusions and finishes with questions for reflection and discussion. This makes it an excellent resource for anyone teaching not only family sociology but more broadly population studies or social sciences. The authors successfully incorporate recent developments into the discussion, from the rise in digital inequalities and challenges of “media parenting”, to the latest findings related to the Covid-19 pandemic. They also highlight how the notion of a decline in traditional family values has been increasingly used by politicians (i.e. by recent US presidents and UK prime ministers) in their election campaigns. As part of this discussion, the authors emphasize the dangers associated with the rise of right-wing populist and conservative views on gender and family, including the recent bans on abortion in Poland and the US and surge in anti-immigrant sentiment.

Taken together, the book successfully meets its aims and is written in an eloquent way, which makes it an easy read. I highly recommend reading this book and including it as recommended reading for students. With that said, I think a few elements could be included in future editions. First, there is very little discussion on heterogeneity in pathways to union and family formation including the increased complexity behind the transition to adulthood. Second, despite its increasing prevalence and heterogeneity in pathways into it, childlessness is only mentioned in the context of ageing. Related to this, the book would also benefit from a broader discussion on the evolution of fertility intentions across the life course.
